# [Corrigendum] Emodin induces apoptosis of human breast cancer cells by modulating the expression of apoptosis-related genes

**DOI:** 10.3892/ol.2026.15892

**Published:** 2026-10-02

**Authors:** Cong Zu, Mingdi Zhang, Hui Xue, Xiaopeng Cai, Lei Zhao, Anning He, Guangyuan Qin, Chunshu Yang, Xinyu Zheng

Oncol Lett 10: 2919–2924, 2015; DOI: 10.3892/ol.2015.3646

Following the publication of the above paper, an interested reader drew to the authors' attention that, regarding the cell invasion assay data shown in Fig. 4 on p. 2922, the ‘Bcap-37/Control’ and ‘ZR-75-30/10 μM’ data panels contained an overlapping section of data, suggesting that these data had been derived from the same original source, even though the results from differently performed experiments were intended to have been portrayed.

Following a thorough re-examination of their raw experimental data, the authors have realized that an inadvertent error was made during the compilation of the data in Fig. 4; specifically, the panel for the ZR-75 cell line had been incorrectly selected from a large collection of original image files. The revised version of Fig. 4, which now includes the correct data for the ZR-75-30/10 μM’ data panel, is shown below.

Note that the replacement of this data panel does not have significant impact on the conclusions reported in this study. All the authors agree with the publication of this corrigendum, and they are grateful to the Editor of *Oncology Letters* for allowing them the opportunity to publish this. The authors also apologize to the readership for any confusion caused.

## Figures and Tables

**Figure 4.**
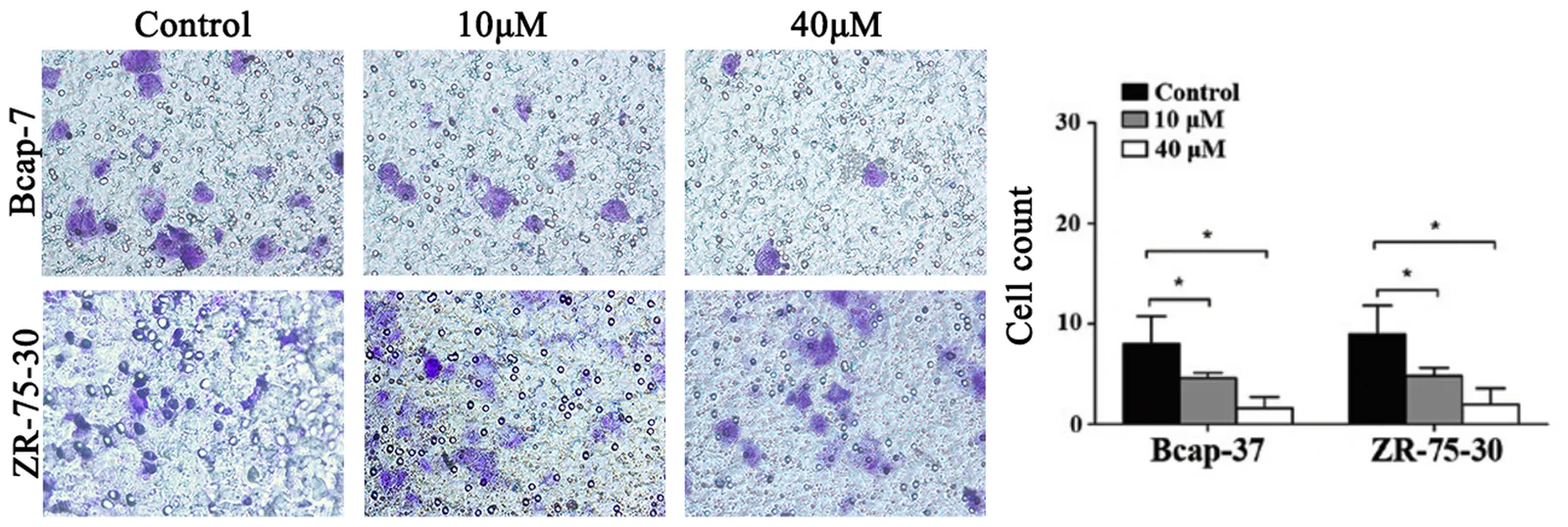
Effects of emodin on invasion of Bcap-37 and ZR-75-30 cells. *P<0.05 vs. control..

